# Supplementary material for: Novel Driver Strength Index highlights important cancer genes in TCGA PanCanAtlas patients
Source: PeerJ. 2022 Aug 11;10:e13860. doi: 10.7717/peerj.13860 (PMC9375969; doi:10.7717/peerj.13860)
Supplement: Supplemental Information 2 [file peerj-10-13860-s002.zip › COHORTS/genes plots/cohorts/2021_8_16_14_9_distribution_cohorts_females_SARC.pdf]

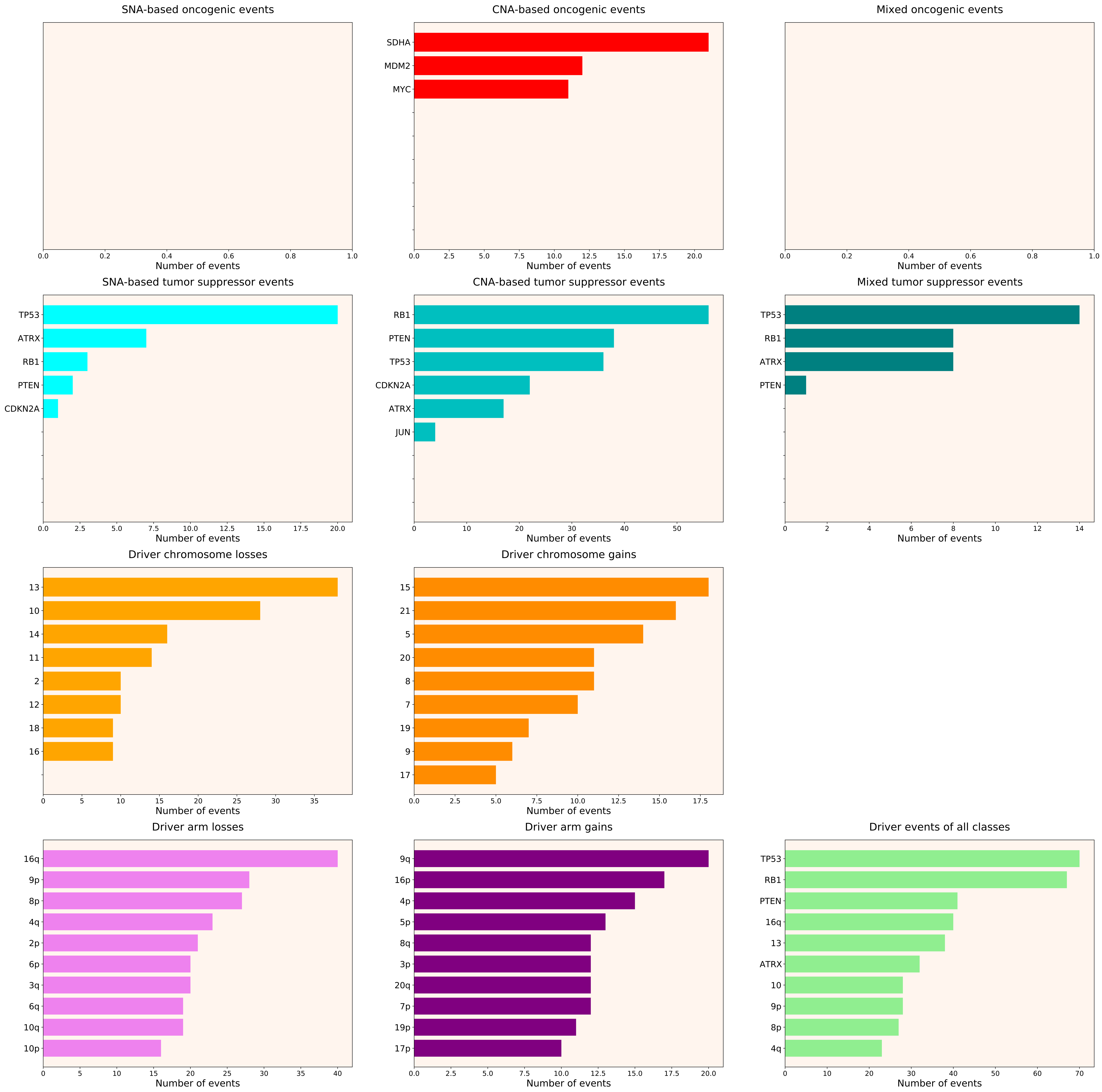
SNA-based oncogenic events
0.0 0.2 0.4 0.6 0.8 1.0
Number of events
CNA-based oncogenic events
SDHA
MDM2
MYC
0.0 2.5 5.0 7.5 10.0 12.5 15.0 17.5 20.0
Number of events
Mixed oncogenic events
0.0 0.2 0.4 0.6 0.8 1.0
Number of events
SNA-based tumor suppressor events
TP53
ATRX
RB1
PTEN
CDKN2A
0.0 2.5 5.0 7.5 10.0 12.5 15.0 17.5 20.0
Number of events
CNA-based tumor suppressor events
RB1
PTEN
TP53
CDKN2A
ATRX
JUN
0 10 20 30 40 50
Number of events
Mixed tumor suppressor events
TP53
RB1
ATRX
PTEN
0 2 4 6 8 10 12 14
Number of events
Driver chromosome losses
13
10
14
11
2
12
18
16
0 5 10 15 20 25 30 35
Number of events
Driver chromosome gains
15
21
5
20
8
7
19
9
17
0.0 2.5 5.0 7.5 10.0 12.5 15.0 17.5
Number of events
Driver arm losses
16q
9p
8p
4q
2p
6p
3q
6q
10q
10p
0 5 10 15 20 25 30 35 40
Number of events
Driver arm gains
9q
16p
4p
5p
8q
3p
20q
7p
19p
17p
0.0 2.5 5.0 7.5 10.0 12.5 15.0 17.5 20.0
Number of events
Driver events of all classes
TP53
RB1
PTEN
16q
13
ATRX
10
9p
8p
4q
0 10 20 30 40 50 60 70
Number of events
